# Trends in prevalence and antibiotic susceptibility of multidrugs-resistant Carbapenem-Resistant *Acinetobacter baumannii* (CRAb) and carbapenem-resistant *Pseudomonas aeruginosa* (CRPa) in blood culture

**DOI:** 10.1017/ash.2025.75

**Published:** 2025-09-03

**Authors:** Andaru Dahesihdewi, Dian Febriyani

**Affiliations:** Departement of Clinical Pathology and Laboratory Medicine Sardjito General Hospital, Faculty of Medicine, Public Health and NursingUniversitas Gadjah Mada, Yogyakarta.

**Keywords:** CRAb, CRPa, MDRO, antibiotic susceptibility, difficult to treat

## Abstract

**Background:** Carbapenem-resistant *Acinetobacter baumannii* (CRAb) and carbapenem-resistant *Pseudomonas aeruginosa* (CRPa) are critical priority MDROs. They can develop resistance to last-line antibiotics, complicating infection treatment, leading to longer hospital stays, higher costs, and mortality. **Objectives:** To describe trends in CRAb and CRPa prevalence and antibiotic susceptibility in the access, watch, and reserve groups of positive blood cultures. **Methods:** This was a descriptive observational study using data from positive blood cultures at Dr Sardjito General Hospital from 2020-2023. Bacterial identification and antibiotic susceptibility testing were performed using the Vitec-2 Compact system. Patient demographics and clinical data were obtained from the microbiology LIS and electronic medical recods. **Results:** A total of 3603 positive blood cultures were obtained. CRAb rates were 85%, 62%, 68%, 78%, while CRPa were 26%, 21%, 12%, 25% respectively. CRAb antibiotic susceptibility in access group: 2%, 8%, 5%, 3%, in watch group: 27%, 28%, 27%, 22% and in reserve group: 33%, 58%, 37%, 30%. CRPa susceptibility was reduced in all groups: 13%, 30%, 13%, 6% in access, 0%, 4%, 0%, 19% in watch, and 13%, 7%, 13%, 14% in reserve group. CRPa was more susceptible in the access group, while CRAb was more susceptible in the watch and reserve groups suggesting that CRPa infections were more difficult to treat. The highest prevalence was in 2020, possibly due to failure to control antibiotic use during the early Covid-19 pandemic. A decrease in both MDR pathogens in 2021 was associated with intense ASP activities in the second year of the pandemic. Increasing prevalence in the following years may be due to a lack of stewardship following a change in the internal antimicrobial stewardship team structure. **Conclusions:** These data indicate that consistent ASP had important role in controlling CRAb and CRPa. Changing the structure of antimicrobial stewardship team should be well prepared to ensure a good adaptation.

